# Testing the coordination hypothesis: incompatibilities in aggregative development of an experimentally evolved social amoeba

**DOI:** 10.1093/evlett/qrae063

**Published:** 2024-11-20

**Authors:** Israt Jahan, Trey J Scott, Joan E Strassmann, David C Queller

**Affiliations:** Department of Biology, Washington University in St. Louis, One Brookings Drive, St. Louis, MO, United States; Department of Biology, Washington University in St. Louis, One Brookings Drive, St. Louis, MO, United States; Department of Biology, Washington University in St. Louis, One Brookings Drive, St. Louis, MO, United States; Department of Biology, Washington University in St. Louis, One Brookings Drive, St. Louis, MO, United States

**Keywords:** aggregative multicellularity, coordination, incompatibilities, experimental evolution, *Dictyostelium discoideum*

## Abstract

Multicellular organisms that form by aggregation of cells arguably do not achieve high levels of complexity. Conflict among the cells is a widely accepted explanation for this, but an alternative hypothesis is that mixing cells of different genotypes leads to failures of coordination, which we call the “coordination hypothesis.” We empirically tested the coordination hypothesis in the social amoeba *Dictyostelium discoideum*. We mixed *D. discoideum* clones that had evolved in isolation for generations and acquired mutations that have not been tested against each other by selection. To quantify the effect of incompatibilities, we measured performance in terms of the developmental traits of slug migration and spore production. Importantly, we mixed lines evolved from the same ancestor under conditions that would not select for the evolution of de novo kin recognition. Our results show no evidence of incompatibilities in four traits related to the coordinated movement of slugs toward light in the social amoeba. Spore production was higher than expected in mixtures, in apparent contradiction to the coordination hypothesis. However, we found support for coordination incompatibilities in an interaction between migration and spore production: in mixtures, fewer cells succeeded at both migrating and becoming spores.

## Introduction

Many familiar life forms have evolved from groups of smaller, formerly free-living biological entities (Bell & Mooers, 1997; Bourke, 2013; Corning & Szathmáry, 2015; Gardner & Grafen, 2009; Orr, 2000; Szathmáry & Maynard Smith, 1995). The process of groups evolving into a higher-level entity upon which natural selection can act is called a major transition. The evolution of multicellularity is one such major transition where single-celled ancestors evolved to take advantage of group living. Early multicellular life set the stage for a new type of biological individual, allowing natural selection to operate at levels previously absent (Clarke, 2016; Díaz-Muñoz et al., 2016; Michod, 2005, 2007; Queller & Strassmann, 2009). How and why multicellular organisms developed and subsequently evolved, diversified, and increased in complexity, is an important question for understanding major transitions.

Multicellularity originated independently at least 20 times across all major domains of life (Grosberg & Strathmann, 2007). Eukaryotic multicellular organisms largely reproduce by undergoing single-cell bottlenecks. For example, sexually reproducing organisms produce gametes that combine into a unicellular zygote. Asexual plants and animals, such as the Amazon molly *Poecilia formosa* (Turner et al., 1980), several weevils (Suomalainen & Saura, 1973), and many angiosperms (Bicknell & Koltunow, 2004) also reproduce by single-cell propagules.

In many organisms, alternative modes of reproduction exist where the offspring is likely to develop from more than one genotype. This type of reproduction may involve simple budding or fission (Åkesson et al., 2001; Galliot, 2012), or specialized structures, such as the fungal conidia or gemmae found in algae, mosses, and ferns (Hughes, 1971). Because the propagules are multicellular, they may include genetic variation that would be passed on to progeny. An extreme form of such chimeric multicellularity is perhaps best demonstrated by the aggregation of individual cells that reside as neighbors (Jahan et al., 2022), known as aggregative multicellularity. The diversity of routes to simple multicellular development raises a fundamental question for the study of organismal complexity: Why haven’t these other modes of multicellularity like aggregation equally contributed to complex development?

Single-cell bottlenecks are thought to be a requirement for complex multicellularity (Fisher et al., 2020; Howe et al., 2024; Wolpert & Szathmáry, 2002). The accepted explanation is kin selection (Axelrod & Hamilton, 1981; Bonner, 1988; Fisher et al., 2013; Hamilton, 1963). Development from a single-cell bottleneck results in a multicellular body with cells that are essentially genetically identical, minimizing genetic conflict and maximizing cooperation between cells within a clonal organism. When an exploiter mutant arises within the multicellular body during development, it might gain an initial advantage. But after a single-cell bottleneck, the mutant will exist in bodies with all mutant cells, and therefore unable to be exploited further. A single-celled propagule thus reestablishes genetic uniformity for the germline, such that exploiters can exploit only during the lifespan of a single organism.

Wolpert and Szathmáry (2002) proposed an alternative (but not mutually exclusive) explanation for why complex multicellular organisms develop from a single cell. They suggested that the development of complex multicellularity is not only a question of kin selection controlling conflict between cells but also an issue of how cells coordinate developmental processes in the organism. For example, the authors suggest cells in chimeric mixtures could fail to generate repeatable developmental patterns:

“…patterning processes require signaling between and within cells, leading ultimately to gene activation or inactivation. Such a process can lead to reliable patterns of cell activities only if all the cells have the same set of genes and obey the same rules.”

They add that this would hinder the evolution of novelty:

“We consider it practically impossible to have several-to-many asexual, partly differentiated, cell lineages mutating in all sorts of directions in genetic space and yet keep up the ability to evolve into viable novel forms.”

We call this idea the “coordination hypothesis.” This hypothesis remains to be tested empirically despite its important implications for the evolution of multicellular development. The lack of empirical tests of the hypothesis can be attributed to two related issues. First, Wolpert and Szathmary are not very explicit about testable predictions. Second, it is difficult to choose a suitable system for experimental manipulation and testing.

Though Wolpert and Szathmary’s ideas may be more complex than our interpretation, we take the coordination hypothesis to include at least the following predictions. First, aggregative development puts new mutations into many new and evolutionarily untested cellular combinations. When this is so, we predict that mixing of cells from different lineages will usually be detrimental for the same reasons that untested mutations are generally detrimental. Second, even if a new advantageous mutation would be beneficial when fixed, to achieve fixation it must be advantageous across many frequencies and many combinations where the mutant cells are located in the organism. Even if some combinations were beneficial, evolution would be impeded if it had to pass through any stage with disadvantageous combinations. Specifically, we predict that this could occur when intermediate mixtures—groups where only some cells have a beneficial mutation—would have lower fitness than when it is present in all cells or none, that is when mixing produces transgressively lower fitness. In contrast, organisms with a single-cell bottleneck do not face these problems because each organism is genetically uniform, and the mutation will fix under the single condition that it is beneficial when present in all cells in an organism.

To make this logic more concrete, consider how evolution might work on human bodies if they were formed by aggregation of diverse cells. A new mutation might find itself first in the liver, but in the next generation, it might find itself in the hypothalamus of one individual, the adenoids of another, and the germ cells of a third. To rise to higher frequencies, it would have to be advantageous, on average, across all these contexts. If the frequency did increase, it would still find itself in more localities and combinations of localities. The complexity increases much more if there are other segregating mutations.

**Figure 1.**
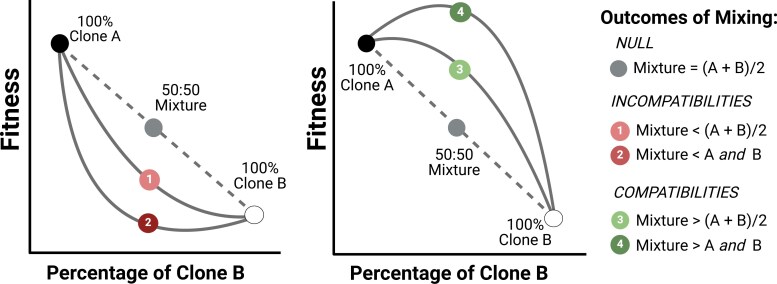
Illustration showing the possible fitness outcomes of mixing between two cell lineages. The gray dotted line represents the null hypothesis (additivity). The panel on the left shows the two predictions of the coordination hypothesis, where incompatibilities in development may impede fitness improvement. The panel on the right shows the possibility of mutations that are compatible and may generate beneficial fitness consequences upon mixing. Circles represent the 50:50 mixtures that will be tested in this study. Created with Biorender.com. Available at https://BioRender.com/f11h199.

In Figure 1, we present a schematic diagram of possible outcomes when cells from distinct lineages are mixed together. The null hypothesis (Figure 1, gray dotted line) predicts that mixing is neither beneficial nor detrimental to multicellularity, such that the fitness of the mixture is the average of the fitness of the two lineages A and B (shown by the gray circle in both panels). This would not impede evolution from the low-fitness B to the high-fitness A. The coordination hypothesis predicts that mixing will have detrimental fitness consequences in the resulting multicellular group (Figure 1, line 1) on average compared to the null expectation. In some extreme cases (Figure 1, line 2), incompatibilities between cells may result in large fitness costs for the mixture causing it to have transgressively lowest fitness. This would imply that a clone starting with a lower fitness (as is the case for clone B) would not be able to evolve to the higher-fitness A state because it would require passing through a lower fitness stage. Under this scenario, intermediate cell combinations would hinder the evolution of increased complexity, as Wolpert and Szathmary argued.

Alternatively, mixing could sometimes result in improved fitness in the mixtures compared to the expected null (Figure 1, lines 3 and 4). Under some conditions, for example, social heterosis (Nonacs & Kapheim, 2007), mixing may generate combinations that are complementary in nature such that each lineage compensates for genetic defects in the other and results in higher or even transgressively higher fitness in the mixture. Though social heterosis is often defined for mixtures of multicellular individuals, such as ants, such complementarity can also apply to microbes (Kraemer & Velicer, 2014). Under social heterosis, mixing is predicted to be favored by natural selection, other things being equal (Ebrahimi & Nonacs, 2021; Nonacs, 2017).

To test the coordination hypothesis, we require a biological system that can be modified to (1) develop from mixtures of genetically different cells (chimeric development), (2) provide developmental mutations for mixing that have not been previously tested by selection, and (3) exclude the alternative hypothesis that incompatibilities are due to adaptive conflicts mediated by kin recognition. The social amoeba *Dictyostelium discoideum* can satisfy these criteria and therefore is an ideal system for this experiment.

**Figure 2.**
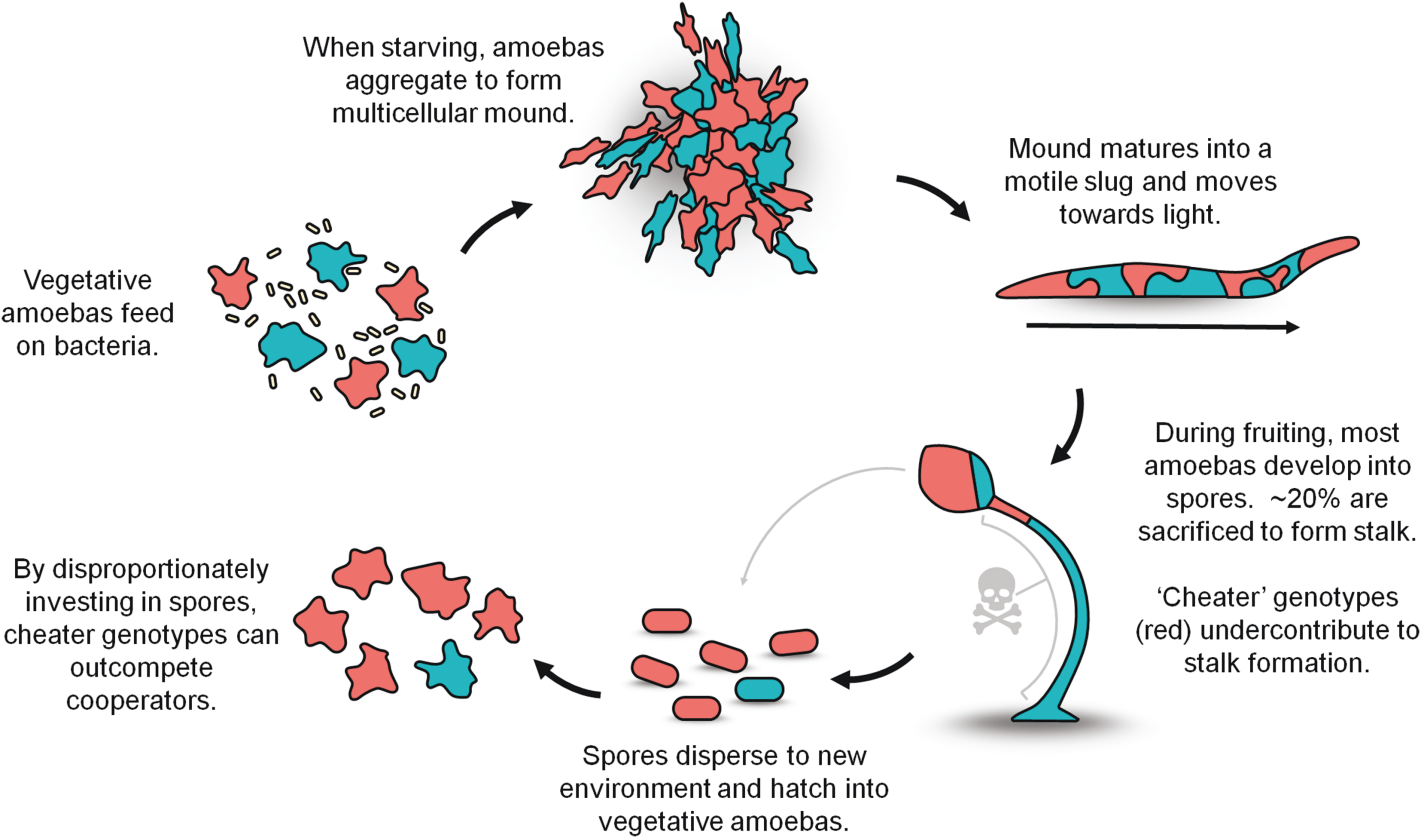
Social life cycle of *D. discoideum* illustrating clone mixing. Life stages are not drawn to scale. Credits Tyler Larsen, CC BY-SA 4.0, via Wikimedia Commons.

Individual *D. discoideum* amoebae divide and grow independently in the presence of abundant edible bacteria but under starvation, they aggregate and undergo multicellular development (Figure 2). At one stage in development, the multicellular aggregate forms a slug that shows coordinated movement toward light. Migrating toward light can assist *D. discoideum* in moving toward the soil surface (Castillo et al., 2005) and increases the chance of spore dispersal by insects, other small invertebrates, and birds (Huss, 1989; Smith et al., 2014). The slug culminates into a fruiting body with some cells becoming spores held atop a slender stalk composed of dead cells. Because of this lifecycle, *D. discoideum* has been extensively used to study chimeric development (Castillo et al., 2011; Chattwood & Thompson, 2011; de Oliveira et al., 2019; Foster, 2010; Khare et al., 2009; Khare & Shaulsky, 2006; Medina et al., 2019; Mehdiabadi et al., 2006; Sathe et al., 2010; Strassmann, 2016; Strassmann & Queller, 2011a, b; Thompson & Kay, 2000).

There are some documented benefits and costs of mixing with nonrelatives (Fortunato et al., 2003; Foster et al., 2002; Strassmann et al., 2000). The known benefit of chimeric development is increased group size. Mixing with nonrelatives can result in larger slugs which can migrate greater distances (Foster et al., 2002). However, when started with the same number of cells chimeric slugs move shorter distances than clonal slugs (Castillo et al., 2005; Foster et al., 2002). Chimeras also produce more spores, which superficially appears to increase fitness but may actually decrease it by reducing stalk production (Belcher et al., 2022; Buttery et al., 2009).

These outcomes could be explained by the coordination hypothesis, but they could also reflect evolved conflict strategies. To test the coordination hypothesis, we therefore need to exclude any evolved antagonistic strategies, which would depend on kin recognition. At least in the early stages of aggregation, a matching pair of cell surface proteins encoded by the *tgrB1* and *tgrC1* loci is necessary and sufficient for attractive self-recognition in *D. discoideum* (Hirose et al., 2011, 2015; Ho et al., 2013). Amoebas can selectively bind to other cells with compatible *tgr* alleles such that a mismatch can cause poor cell-cell adhesion, and even assorting into individual clones (Benabentos et al., 2009; Hirose et al., 2011, 2015, 2017; Ho et al., 2013; Ostrowski et al., 2008), although sorting may largely disappear at later stages, perhaps due to slug fusion (Gilbert et al., 2012).

Evolving different *D. discoideum* strains from a common ancestor can give us genetically different clones for mixture experiments. If these strains evolve without the multicellular social stage, there can be no direct selection or adaptation for social traits, including kin recognition (de Oliveira et al., 2019). In a recent experimental evolution study (Larsen et al., 2023), we created such lineages of *Dictyostelium discoideum* under relaxed (zero) selection for collective behavior and aggregation of cells. Amoebas were transferred on nutrient plates before the onset of starvation, for 30 transfers (more than 200–300 cell divisions). Though there was no direct selection for social traits, these lines evolved altered social behaviors via drift or pleiotropy with traits under selection; they showed reduced cheating, slug migration, and increased spore production. In sum, we have experimental lines that have not adapted to conflict with each other but differ genetically in social traits that have never been selectively tested in combination. Here we use these experimental lines to generate chimeric mixtures and test for reduced coordination in slug migration and spore production.

## Materials and methods

### Chimeric mixes

Our study involved two kinds of mixtures: pairwise and complex. First, we made pairwise mixes of clonal ancestors with one of their evolved lineages. We used 10 *D. discoideum* ancestors (QS6, QS9, QS11, QS18, QS69, QS70, QS159, QS161, QS395, and QS859) that had been experimentally evolved in triplicate to generate 30 evolved lines in total (Larsen et al., 2023). We made pairwise mixes of the ancestral clones (A) with each of their own derived lines (E1, E2, and E3) to generate a total of 30 mixed lines (A + E1, A + E2, and A + E3; we call these “lines” for consistency, but they are really mixtures). Therefore, we had a total of 70 experimental lines from 10 *D. discoideum* clones. We had three technical replicate plates for each of the 70 experimental lines resulting in a total of 210 plates for three treatments—ancestor, evolved, and mixed.

To generate a complex mixture, we mixed the three evolved lineages derived from a common ancestor with each other for 3 out of 10 strains (QS6, QS9, and QS18). There should be twice as many differences between two evolved lines as between ancestor and evolved, generating greater power in detecting incompatibilities, should they occur. Mixed lines from complex mixtures did not include the ancestors. We repeated the experiment with complex mixtures on 3 different days to enhance power, resulting in a total of 90 plates for the analyses (30 plates × 3 days). On each day, we had two replicate plates for every “Unmixed” evolved line (E1 or E2 or E3) and four replicate plates for every “Mixed” treatment (equal proportions of E1 and E2 and E3) resulting in a total of 10 plates for every strain.

### Slug migration assay

To obtain spores to inoculate the slug migration assays, we first took glycerol stocks of *D. discoideum* spores stored at −80 °C and streaked each frozen sample on solid SM/5 agar plates (Fey et al., 2007). After development, we collected spores and plated 2 × 10^5^ of them onto a fresh SM/5 plate along with 200 µl *K. pneumoniae* food bacteria resuspended in KK2 buffer (OD600 = 1.5). This round of growth was to remove freezer effects. On day 4, we harvested spores from developed fruiting bodies and initiated the slug migration assay on nonnutrient agar plates (13 cm diameter), in three replicates for each ancestral clone, their evolved lineages, and mixtures.

**Figure 3.**
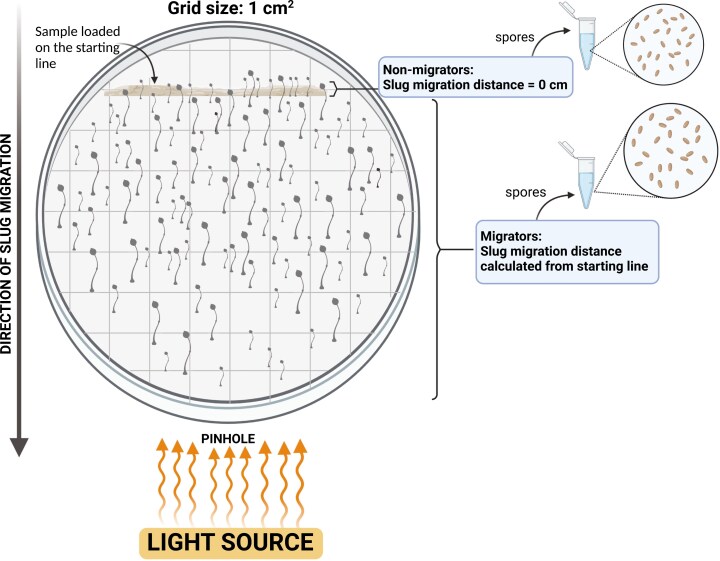
Schematic depiction of an experimental plate at the end of the slug migration assay. We refer to fruiting bodies that developed directly on the inoculation site as nonmigrators and fruiting bodies that developed beyond the inoculation line as migrators. Fruiting bodies on a plate, especially nonmigrators, were more numerous than shown in the diagram. Distance traveled by migrators toward the light source was measured from the inoculation line. We counted spores from both migrator and nonmigrator fruiting bodies and added the two to obtain the total spores produced on a plate. Objects not drawn to scale. Created with BioRender.com. Available at https://BioRender.com/a53i933.

On every plate, we marked a 10 cm secant line (henceforth inoculation line) on which we loaded spore samples (Figure 3). For pairwise mixtures, we loaded a sample consisting of 10^7^  *D. discoideum* spores in 50 μl of *K. pneumoniae* (OD600 = 50.0) suspended in KK2 buffer. The “mixed” treatment for pairwise mixtures was made of 50% of evolved (E1 or E2 or E3) and 50% of ancestor (A) spores in the total 10^7^-spore suspension. For samples of complex mixtures, we loaded 9 × 10^6^ spores suspended in 50 μl of *K. pneumoniae* food bacteria (OD600 = 50.0) on the inoculation line as in pairwise mixtures. Spores plated at low densities can give rise to clonal patches and unmixed aggregates, but the density of spores plated here is orders of magnitude higher than that required to avoid this effect and get equal mixing (Smith et al., 2016).

After loading the suspension along the starting line of the agar plates, we allowed the sample to dry and wrapped the plates individually in aluminum foil. We made a small pinhole opposite the starting line on each wrapped plate. For the duration of the experiment, light enters only through this small pinhole toward which slugs migrate from the other end of the plate. We then left the wrapped plates undisturbed for 8 days with the pinhole side of the plate facing a light source in the laboratory. Because the plates had no nutrients, bacteria and amoebas grew only at the starting line, but slugs could migrate off the starting line toward the light. We unwrapped the plates at the end of the 8 days and allowed fruiting bodies to finish developing fully under direct light.

### Image analysis

We used a Canon EOS 5D Mark III camera to photograph each plate. We put each plate on a laboratory bench with the camera mounted at a fixed distance. We obtained slug migration distances using the software Fiji and ImageJ (Bourne & Bourne, 2010; Rueden et al., 2017; Schindelin et al., 2012). We processed each image by first scaling it and then overlaying a 1 cm × 1 cm grid. We recorded the distance of each fruiting body from the starting line. For pairwise mixes, we assigned distance = 0 cm for fruiting bodies that developed directly on the inoculation line and referred to those as “nonmigrators.” Fruiting bodies that traveled beyond the inoculation site are referred to as “migrators” (Figure 3).

We observed that a few slugs traveled in the opposite direction to light. In the follow-up complex-mixture experiment, to account for such slugs that may contribute to a reduced average migration distance of slugs on a plate, we measured the distances of all fruiting bodies from the inoculation line on a plate. Our measurements for complex mixtures therefore account for positive or negative movement toward light for slugs on a plate.

### Spore production assay

At the end of the slug migration assay for pairwise mixtures, we quantified the number of spores produced on a plate by slugs that migrated toward light as well as those that did not leave the inoculation site. We collected nonmigrators and migrators for each plate in separate eppendorf tubes containing 1 ml KK2 buffer. We made 1:100 dilutions of the collected spores and counted them using a hemacytometer. We added the number of spores produced by migrators and nonmigrators to obtain the total spore production on a plate for each strain across three treatments. We performed a log transformation for use in our analyses. This assay was performed only for pairwise mixtures and not for complex mixtures.

### Statistical analysis

We used R version 4.2.1 (R Core Team, 2022) for all our analyses. We used the *tidyverse* (v2.0.0) suite of packages for data cleaning, and the package *fitdistrplus* (v1.1.11) for fitting univariate distributions to our data (Delignette-Muller & Dutang, 2015). For both pairwise mixtures and complex mixtures, we performed linear mixed effects modeling with the *lme4* (v1.1.35.2) package (Bates et al., 2015). Treatment and clone were fixed effects and replicate experimental line as random effect for the analysis of both slug migration and spore production data. Pairwise mixtures included three treatments (ancestor, mixed, and evolved) whereas complex mixtures included two treatments (mixed and unmixed). We used Akaike information criteria for model selection which estimates the relative quality of each model based on model quality and parsimony. To assess model fit and assumptions, we used the package *performance* (v0.11.0) (Lüdecke et al., 2021).

We fitted a generalized linear mixed effects model to our proportion data from pairwise mixtures using the package *glmmTMB* (v1.1.9) with a beta-binomial distribution (Brooks et al., 2017). For the analysis of slug migration distances, we used the *robustlmm* (v3.3.1) package (Koller, 2016) to account for outliers without their removal from the data used in our final model. This procedure prevents outliers from unduly influencing estimations by inversely weighing them. We reported estimated marginal means using the *emmeans* package (v1.10.5) (Lenth, 2024) averaged at the level of clone for all the statistical models. We performed hypothesis testing using estimated marginal means and inferred the statistical significance of our results after adjusting for multiple hypothesis comparisons for false discovery rates. All graphs in the results section were plotted with the *ggplot2* (v3.5.1) package and significance values were displayed using the *ggsignif* (v0.6.4) package.

## Results

### No lack of coordination for fruiting body number and slug migration

**Figure 4.**
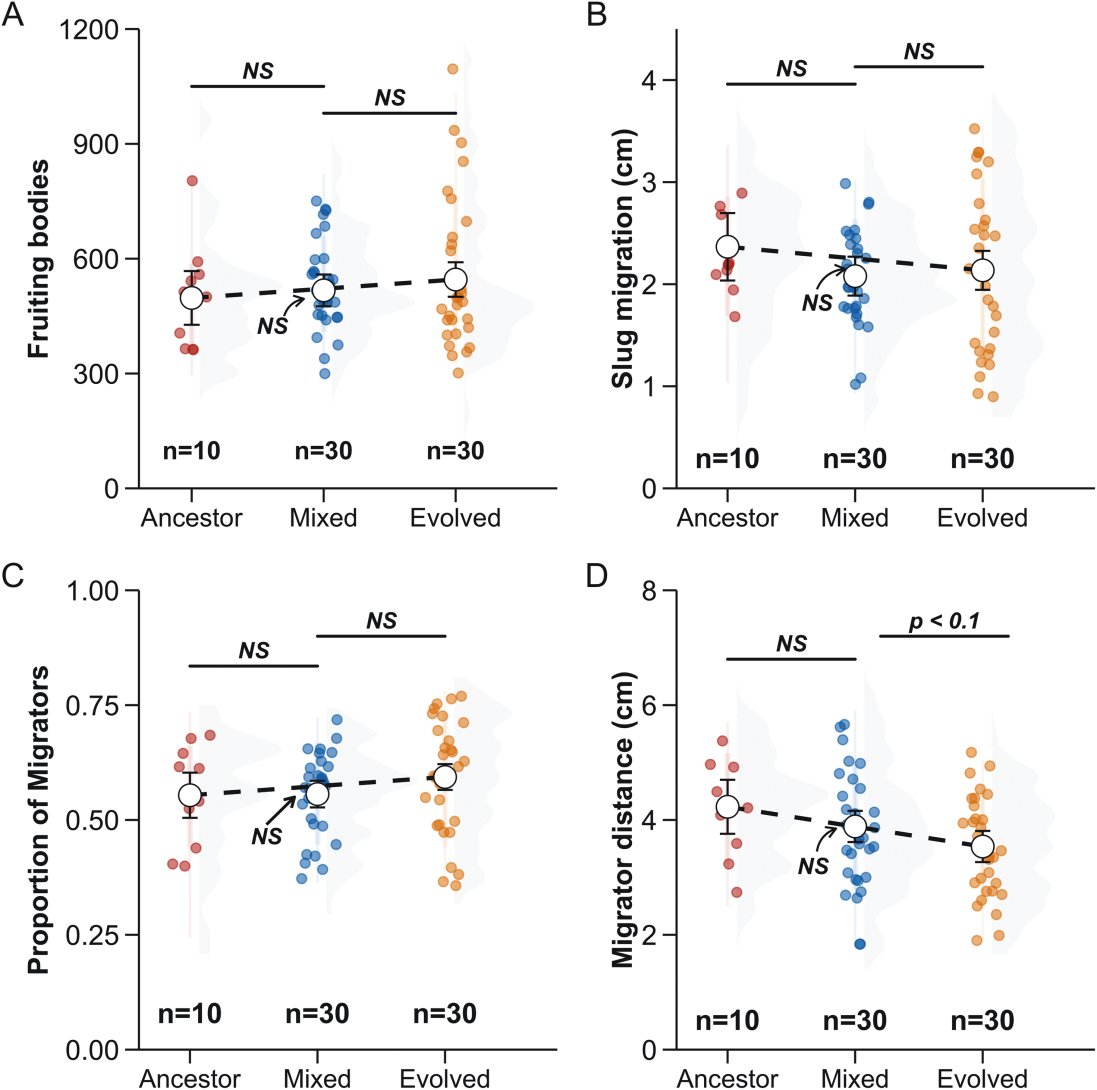
Traits associated with slug migration distance in pairwise mixtures of *D. discoideum* ancestral strains and their evolved descendants. There is no significant difference between the expected average of the ancestor and evolved cell lines and observed values in mixtures for any of these traits: (A) number of fruiting bodies (migrators + nonmigrators), (B) slug migration distance, (C) proportion of migrators, and (D) distance traveled by migrators. The expected averages of pairwise mixtures between ancestor and evolved lines are depicted with a dashed line, against which the observed mixed point is compared. Error bars correspond to 95% confidence intervals of the estimated marginal means, which are shown with the white central points.

We quantified the outcome of mixing on slug migration in 10 strains of *D. discoideum*. First, we created pairwise mixtures between clonal ancestors and their experimentally evolved descendants for the three treatments: ancestors, evolved, and mixed (50% ancestor + 50% evolved). We quantified the total number of fruiting bodies on a plate and the proportion of them that had migrated toward light (Figure 4A and B). There was no significant difference between the expected and the observed values of total number of fruiting bodies on a plate ([ancestor + evolved]/2 − mixed = 4.32, *SE* = 24.6, *z* ratio = 0.176, *p* value = 0.8606; Figure 4A). The proportion of migrators on a plate was not significantly different either ([ancestor + evolved]/2 − mixed = 0.01735, *SE* = 0.0169, *z* ratio = 1.026, *p* value = 0.4571; Figure 4C).

We measured average slug migration distance for each treatment which included values from both migrators and nonmigrators. For pairwise mixtures, chimeras were not significantly different from the expected value on average ([ancestor + evolved]/2 − mixed = 0.172, SE = 0.113, *z* ratio = 0.1528, *p* value = 0.19), as shown in Figure 4B. We then compared slug migration distances for only the migrators for the three treatments. Mixed lines were extremely close to the expected value ([ancestor + evolved]/2 − mixed = −0.00346, *SE* = 0.16, *z* ratio = −0.022, *p* value = 0.9828; Figure 4D).

**Figure 5.**
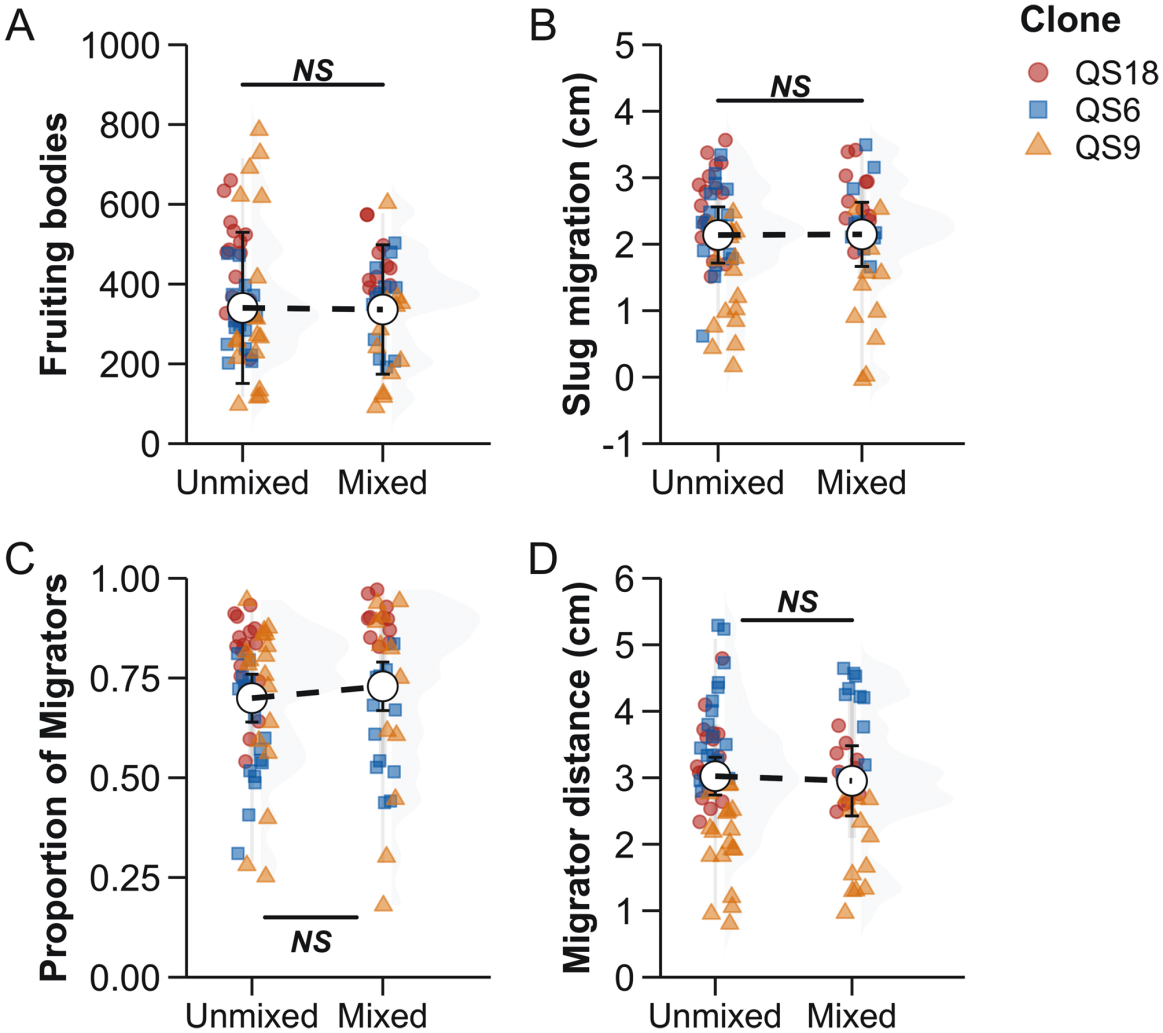
Traits associated with slug migration in complex mixes of evolved lineages of a clonal *D. discoideum* ancestral strains. There is no significant difference between the expected average of the evolved cell lines and observed values in mixtures for (A) number of fruiting bodies (migrators + nonmigrators), (B) average slug migration distance, (C) proportion of migrators, and (D) distance traveled by migrators on average. Each point on a graph represents the average of three replicate plates for a strain. Data are averaged at the level of clones (QS6, QS9, and QS18), shown with different shapes and colors.

We performed a second slug migration experiment with the aim of getting increased power to detect any incompatibilities. We mixed three descendants (from the same ancestor) to obtain a more complex mixture that should allow greater statistical power and thus robust inference. We expect the mixture of three evolved lines to include more genetic differences than the pairwise mixes, for two reasons. First, more cell variants are mixed together. Second, two descendants have twice as many differences as ancestor and descendant. Our analysis comparing unmixed and mixed lineages of a clonal ancestor still showed no significant differences in any of the four traits: total number of fruiting bodies on a plate (unmixed − mixed = 15.5, *SE* = 40.7, *z* ratio = 0.381, *p* value = 0.7032; Figure 5A), the average slug migration distances (unmixed − mixed = −0.00976, *SE* = 0.197, *z* ratio = −0.050, *p* value = 0.9604; Figure 5B), proportion of migrators on a plate (unmixed − mixed = −0.0296, *SE* = 0.0296, *z* ratio = −0.999, *p* value = 0.3176; Figure 5C), distance travelled by migrators on a plate (unmixed − mixed = 0.0424, *SE* = 0.15, *z* ratio = 0.283, *p* value = 0.7773; Figure 5D).

### Transgressively fewer cells in mixtures can both migrate and produce spores

**Figure 6.**
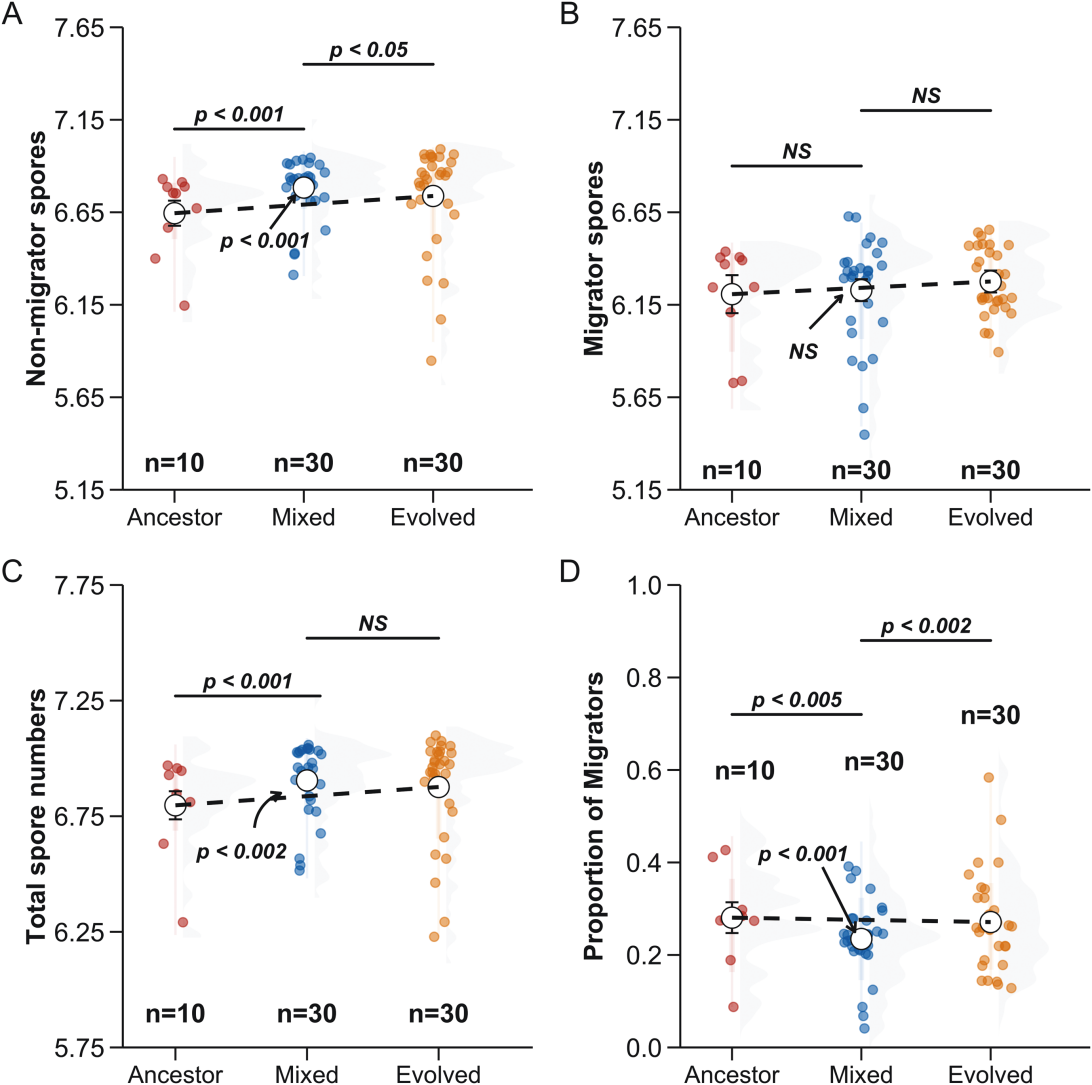
Spore production (estimated as log_10_ transformed spore numbers) in pairwise mixtures is significantly different from *D. discoideum* ancestral strains and their evolved descendants depending on whether spores were produced by migrators fruiting bodies. (A) Nonmigrator fruiting bodies produce a transgressively higher number of spores in mixtures, (B) migrator spores are not significantly different across treatments, (C) mixtures produce a higher number of total spores (migrators + nonmigrators) than the expected average, and (D) mixed migrators produce a transgressively lower proportion of spores. The expected averages of pairwise mixtures between ancestor and evolved lines are depicted with a dashed line, against which the observed mixed point is compared. Error bars correspond to 95% confidence intervals of the estimated marginal means, which are shown with the white central points.

At the end of migration, cells in slugs can get into the next generation by successfully producing spores. We measured the number of spores produced by developed fruiting bodies from the pairwise mixes at the end of the slug migration assay for both migrator as well as nonmigrator fruiting bodies and obtained total spore production on a plate by adding the two values. We performed a log_10_ transformation to the observed spore numbers for statistical comparisons and data visualization (Figure 6).

First, we compared the number of spores produced by nonmigrators across the three treatments. Nonmigrator fruiting bodies produced significantly more spores in mixtures than the expected average of their constituent ancestors and evolved strains ([ancestor + evolved]/2 − mixed = −0.0920, *SE* = 0.0231, *z* ratio = −3.977, *p* value = 0.0001; Figure 6A). Nonmigrator spore numbers in mixtures were also significantly greater than both ancestral as well as evolved lines (ancestors − mixed = −0.1385, *SE* = 0.0327, *z* ratio = −4.233, *p* value = 0.0001, evolved − mixed = −0.0455, *SE* = 0.0231, *z* ratio = −1.968, *p* value = 0.0491; Figure 6A).

We then compared spore numbers for migrators on a plate. Interestingly, we found no significant differences in the number of spores produced by fruiting bodies after migrating toward light ([ancestor + evolved]/2 − mixed = 0.0126, SE = 0.0350, *z* ratio = 0.359, *p* value = 0.7194, ancestor − mixed = −0.0217, *SE* = 0.0495, *z* ratio = −0.437, *p* value = 0.7194, evolved − mixed = 0.0468, *SE* = 0.0350, *z* ratio = 1.337, *p* value = 0.5434; Figure 6B).

When total number of spores produced on a plate (migrators + nonmigrators) were compared (Figure 6C), we found that mixed lines, on average, produced significantly more total spores than the expected average of ancestral and evolved lines of *D. discoideum* ([ancestor + evolved]/2 − mixed = −0.1563, *SE* = 0.0477, *z* ratio = −3.272, *p* value = 0.0016). Moreover, the mixed lines produced a significantly higher number of spores than the ancestral lines (ancestor − mixed = −0.2474, *SE* = 0.0675, *z* ratio = −3.664, *p* value = 0.0007). The point estimate was also higher than the evolved lines, hinting at transgressive behavior, but it was not significantly higher (evolved − mixed = −0.0283, *SE* = 0.0207, *z* ratio = −1.364, *p* value = 0.1727).

To further assess the interaction between slug migration and spore production for the ancestors and the evolved lines, we quantified the proportion of spores produced by migrators relative to total spore production on a plate. The proportion of spores produced by mixed migrators was significantly less than the expected average of ancestors and mixed lines ([ancestor + evolved]/2 − mixed = 0.0413, *SE* = 0.0109, *z* ratio = 3.790, *p* value = .0005), as shown in Figure 6D. More striking, mixtures produced a significantly lower number of spores than both the ancestors (ancestor − mixed = 0.0461, *SE* = 0.0158, *z* ratio = 2.925, *p* value = 0.0034), and the evolved lineages (evolved − mixed = 0.0366, *SE* = 0.0108, *z* ratio = 3.386, *p* value = 0.0011). Note that Figure 6D is like Figures 4C and 5C, except that the proportion migrating is now expressed in terms of spores rather than fruiting bodies. Transgressively, fewer spores in mixtures enjoy the benefits of migration.

## Discussion

Complex multicellular life more commonly arises in lineages with single-celled bottlenecks (Fisher et al., 2020; Grosberg & Strathmann, 2007; Howe et al., 2024). This may be because of reduced conflict when the multicellular body consists of close relatives. An alternative explanation, which we call the coordination hypothesis, is that the mixing of genetically distinct cells with different mutations in novel, untried combinations, is expected to have a detrimental effect on the developing multicellular organism due to incompatibilities in coordinating development (Wolpert & Szathmáry, 2002). Instead of a clash of adaptive antagonisms, the coordination hypothesis posits a failure of adaptation due to untested combinations of cell genotypes. The presence of these untested incompatibilities could help explain why aggregative organisms are relatively rare and less complex compared to clonally developing multicellular organisms (Wolpert & Szathmáry, 2002).

To test the coordination hypothesis, we investigated whether mixing experimentally evolved lineages (with ancestors, and among themselves) with mutations that have not been tested against each other by selection would result in detrimental effects in the multicellular stage of an aggregative social amoeba. We predicted detrimental consequences of mixing could manifest in two different ways. First, mixing between two isolated lineages can lower fitness on average. Second, mixtures could have lower fitness than both constituent cell lineages such that mixtures have transgressively lower fitness. We looked for such developmental incompatibilities in *Dictyostelium discoideum* by measuring slug migration distance and spore production.

With respect to slug migration distance, we observed no evidence for incompatibility. We examined four different traits; number of fruiting bodies (which is closely related to number of slugs), the proportion of fruiting bodies that had migrated as slugs, the average migration distance, and the average migration distance for migrators only (Figure 4). For each trait, there was no indication of incompatibility; mixtures were not significantly different from the mean of the ancestor and evolved lines. Because of these negative results, we attempted to increase the power of detecting incompatibilities, making more complex mixtures of three descendant lines of a clonal ancestor. However, these complex mixtures also did not show any evidence of incompatibilities (Figure 5). These results for slug migration fail to provide any support for the coordination hypothesis prediction that previously unselected combinations of genotypes would be deleterious.

Spore production presents a different picture (Figure 6). Mixed nonmigrators not only produced more spores than expected based on the average of nonmigratory ancestors and evolved lineages; they also produced a transgressively higher number of spores (Figure 6A). Interestingly, for migrators, spore neither of these conditions held (Figure 6B). For total spore numbers, we found that mixtures produced a significantly greater number of spores than expected. The result appeared transgressively higher, but was not significantly so, given that mixtures were not significantly higher than the evolved lineages (Figure 6C).

The increase in spore numbers in mixtures from the expected value (Figure 6A and C) may appear to be opposite to the incompatibility hypothesis, if we assume that higher spore production results in higher fitness. Spores are often used as a measure of fecundity (Buttery et al., 2009; Kuzdzal-Fick et al., 2023; Scott et al., 2022). However, an increase in spore production could actually decrease fitness if it is generated at the cost of stalk formation or by producing smaller spores. Counting stalk cell numbers is not feasible but there is considerable evidence to support a spore/stalk trade-off (Belcher et al., 2022). Further support for this possibility comes from the experiment that generated our lines (Larsen et al, 2023). Because there was no social stage during the experimental evolution, we expected most aspects of social fitness to change negatively, and both the ability to cheat and the ability to migrate declined as expected. But spore production increased, suggesting though certainly not proving, that an increase in spore production may represent a decline in fitness.

More convincing evidence for a decline in fitness, including transgressive decline, comes from the interaction between migration and spore number. We could not look at the interaction at the individual slug level because we did not count spore numbers for individual migrating slugs. However, we collected and counted the total numbers of spores for migrating and nonmigrating slugs from pairwise mixtures, and as noted above, they behaved differently (Figure 6A and B).

As a result of this difference, mixtures showed transgressively lower proportions of total spores coming from migrating slugs (Figure 6D) with respect to total spores produced on a plate. Although we saw no significant incompatibility in the proportion of slugs migrating (Figure 4B), there is incompatibility when the migrating proportion is expressed in terms of ultimate spore number (Figure 6D). Fewer spores enjoyed the benefits of migration, and this presumably reflects a fitness cost. Under natural conditions where aggregations can occur below the soil surface, phototactic migration toward the surface can be essential for dispersal and fitness (Castillo et al., 2005). If the production of transgressively lower spores after migration is indeed disadvantageous, then our result supports the argument that incompatibilities in mixtures could prevent evolution from the lower-fitness state to the higher-fitness one.

To demonstrate the incompatibilities due to the coordination hypothesis, we needed to eliminate the alternative explanation for single-cell bottlenecks: adaptive conflict between different clones. In our study, there should have been no kin discrimination since the mixed lines were derived from the same clone. *D. discoideum* does have a recognition system, driven entirely by matching by the products of the *tgrC1* and *tgrB1* loci. Could these loci have evolved via drift or selection? It is extremely unlikely that genetic drift could cause changes in the *tgrB1* and *tgrC1* recognition loci across numerous lines given the relatively short duration of our experimental evolution, the large population sizes, and a very low mutation rate in *D. discoideum* (Kucukyildirim et al., 2020). With respect to selection, *tgrC1* and *tgrB1* are expressed only in the social stages (de Oliveira et al., 2019), and these stages did not occur in the evolving lines (Larsen et al, 2023). There could therefore have been no social cheating during their evolution, and therefore no direct selection for kin recognition cheats or to avoid cheaters. Kin recognition could only have changed by indirect selection on some other trait, but then any incompatibilities would still not be due to *selected* antagonistic kin recognition, so we can still eliminate that hypothesis. Instead, such incompatibilities would be unselected side effects consistent with a failure of coordination among genetic variants that have not been previously selected together.

Data from other studies of chimeric mixtures do not address the coordination hypothesis as clearly because they are likely complicated by antagonistic adaptations and kin recognition. Previous studies in *D. discoideum* have created chimeras by mixing naturally isolated wild genotypes with each other and then compared them to clonal isolates (Castillo et al., 2005; Foster et al., 2002; Jack et al., 2011, 2015; Kuzdzal-Fick et al., 2023). For example, Foster et al. (2002) showed that slug migration distances are lower in chimeras compared to clonal slugs. This would appear to support the coordination hypothesis, but our results suggest that this decreased migration is not due to lack of coordination. But it could be the *tgrB1*/*tgrC1* recognition system, which operates via adhesion cells of the same genotype (Benabentos et al., 2009; Hirose et al., 2011, 2015; Ho et al., 2013, Ostrowski et al., 2008, 2015). Decreased migration could be a selected adaptive competitive effect, with lower adhesion causing cells to drop to the back of the slug, where the cells that become spores are found (Foster et al., 2002). Alternatively, the actual function of the lower adhesion at mismatched *tgr* loci might be to separate the two clones to avoid cheating, with the poorer movement of mixed slugs being a side effect (Khare & Shaulsky, 2010; Khare et al., 2009).

A study quite similar to ours used the social bacterium *Myxococcus xanthus,* which also cooperatively forms multicellular structures. Lines clonally evolved from a common ancestor showed incompatibilities in merging and swarming together when they were mixed (Rendueles et al., 2015). The authors favored the interpretation that these incompatibilities were not directly selected, which would make the results consistent with the coordination hypothesis. However, the evolving *M. xanthus* lines were allowed to socially swarm and form fruiting bodies so one cannot rule out direct selection for cheater resistance.

Rendueles et al. also suggested that, if the incompatibilities were not selected by kin recognition, then they would be very similar to Bateson–Dobzhansky–Muller incompatibilities, which cause isolated populations to be increasingly sexually incompatible and eventually become separate species (Bateson, 2009; Dobzhansky, 1934; Orr, 1996; Orr & Turelli, 2001; Turelli & Orr, 2000). As one population evolves through natural selection, newly emerged genes are tested against existing genes in the population and selected to be compatible with the background. Novel genes that cause mismatches within a population are weeded out, but new genes in one population are never tested with genes that may have newly emerged in another isolated population. Therefore, mismatches between populations gradually accumulate and only become visible to natural selection when the populations later come into contact. For the coordination hypothesis, the incompatibilities must occur between interacting cells, arise within one population, and emerge on a much shorter time scale.

The prevalence of a single-cell bottleneck in multicellular lineages, particularly in complex multicellular organisms, could be due to the importance of high relatedness to cooperation among cells (cooperation) or to the importance of genetic uniformity for coordination. There seems little doubt that kin-selected cooperation is important (Katoh-Kurasawa et al., 2024; Kay et al., 2020; West et al., 2021), but it has been much harder to test the coordination hypothesis, particularly because evolved antagonistic effects are hard to exclude. We excluded them by mixing clonal lines evolved from a single ancestor under nonsocial conditions that should prevent selection for kin recognition and antagonism.

Our results suggest that the coordination hypothesis applies to some traits but not others. The four slug migration traits showed no evidence that mixtures lack coordination. However, results for spore production provide some support for the idea that mixtures of cells may differ from the means of their constituent clones and that this difference is plausibly bad for fitness. For the proportion of spores migrating, the difference is probably bad for fitness, and it is transgressive in a way that could impede evolution.

## Data Availability

The code to reproduce the results and plots in this study is in a GitHub repository (www.github.com/jahanisrat/WolpertHypothesis). Data used for statistical analyses are available in the submitted repository. Raw images used for this study are archived on Zenodo (doi:10.5281/zenodo.12460200).
